# Microbiota in the Natural History of Pancreatic Cancer: From Predisposition to Therapy

**DOI:** 10.3390/cancers15010001

**Published:** 2022-12-20

**Authors:** Cecilia Binda, Giulia Gibiino, Monica Sbrancia, Chiara Coluccio, Maria Cazzato, Lorenzo Carloni, Alessandro Cucchetti, Giorgio Ercolani, Vittorio Sambri, Carlo Fabbri

**Affiliations:** 1 Gastroenterology and Digestive Endoscopy Unit, Forlì-Cesena Hospitals, Ausl Romagna, 47121 Forlì-Cesena, Italy; 2 Department of Medical and Surgical Sciences—DIMEC, Alma Mater Studiorum, University of Bologna, 40138 Bologna, Italy; 3 General and Oncologic Surgery, Morgagni—Pierantoni Hospital, AUSL Romagna, 47121 Forlì, Italy; 4 Microbiology Unit, Hub Laboratory, AUSL della Romagna, 47121 Cesena, Italy

**Keywords:** oral microbiota, pancreatic microenvironment, chronic pancreatitis (CP), autoimmune pancreatitis (AIP), pancreatic cystic neoplasm (PCN), microbiota modulation

## Abstract

**Simple Summary:**

Pancreatic cancer is still burdened with a severe prognosis, despite advances in the diagnosis and surgical management of this disease. The gut microbiome is gaining increasing interest in the development and management in this setting. The intent of our review is to provide a comprehensive review for researchers and clinicians in the field to fully understand the role of the gut microbiome in the history of pancreatic cancer. We analyzed current literature from pre-cancerous conditions to cancer characteristics and how this may alter the therapeutic approach. Evidence and concerns can guide future research in this area.

**Abstract:**

Early microbiome insights came from gut microbes and their role among intestinal and extraintestinal disease. The latest evidence suggests that the microbiota is a true organ, capable of several interactions throughout the digestive system, attracting specific interest in the biliopancreatic district. Despite advances in diagnostics over the last few decades and improvements in the management of this disease, pancreatic cancer is still a common cause of cancer death. Microbiota can influence the development of precancerous disease predisposing to pancreatic cancer (PC). At the same time, neoplastic tissue shows specific characteristics in terms of diversity and phenotype, determining the short- and long-term prognosis. Considering the above information, a role for microbiota has also been hypothesized in the different phases of the PC approach, providing future revolutionary therapeutic insights. Microbiota-modulating therapies could open new issues in the therapeutic landscape. The aim of this narrative review is to assess the most updated evidence on microbiome in all the steps regarding pancreatic adenocarcinoma, from early development to response to antineoplastic therapy and long-term prognosis.

## 1. Introduction

Pancreatic cancer (PC) is an aggressive disease with an increased worldwide incidence. Currently, it is the fourth cause of cancer-related deaths, but it is expected to become the second one by 2030 [1]. Pancreatic ductal adenocarcinoma (PDAC) is the histotype corresponding to approximately 90% of cases of pancreatic malignancy and originates from pancreatic ducts. The current standard of care for patients with PDAC consists of curative surgery, but only 20% of PDACs are diagnosed within resectability criteria [2]. Therefore, the current overall prognosis remains poor (5-year survival at 9%) [3,4].

Only 10% of PDACs are linked to genetic mutations such as BRCA2, STK11/LKB1, CFTR, and PRSS1 or to familial syndromes such as Von Hippen-Lindau disease (VHL), multiple endocrine neoplasia syndrome type 1 (MEN-1), and neurofibromatosis type 1 (NF-1). Accordingly, the majority (90%) of PDACs are sporadic and related to several risk factors such as age, gender, alcohol, smoking, obesity, and lack of physical activity [5]. Furthermore, in the last years, various studies proposed a relationship between the development of PDAC and microbiota imbalance as known for *Helicobacter pylori (Hp)*-gastric adenocarcinoma (GAC) and *Human Papilloma Virus (HPV)*-endometrial adenocarcinoma (EAC). Understanding further pathogenic mechanisms could allow us to change the prognosis of this disease in the future. As is the case of many other diseases, efforts to understand the role of the microbiota have been conducted in the last years [6,7,8,9,10].

The human microbiota consists of the totality of commensals (archeobacteria, bacteria, fungi, and viruses) within our body and above all our gut [11]. This complex structure represents a real continuous changing organ related to multiple factors such as diet, drugs, old age, and even mental status [9,12]. Growing evidence has suggested an independent relationship between microbiota dysbiosis and pancreatic diseases such as chronic and autoimmune pancreatitis (CP, AIP), pancreatic cystic neoplasms (PCNs), and even PDAC [1,13]. Once the pancreas was considered a sterile organ, but in actuality, several studies have established the presence of microbiota within this organ in normal and pathological states although with critical differences [14]. Indeed, microbiota could migrate to the pancreas through the gastroenteric tract or via the mesenteric venous and lymphatic system [15,16].

In addition to the involved microorganisms themselves, there are also several relationships with respect to the molecular contribution encoded by the commensals. Metabolites, derived from the microbiome, could influence molecular processes in cells, including in pancreatic ones. Several mechanisms linked to PDAC oncogenesis have been involved in chronic flogosis promotion through Toll-like receptors (TLRs) and nuclear factor kappa B (NF-kB) cascade activation [4] by lipopolysaccharide (LPS) release. Moreover, polyamines could be able to directly promote the development of PDAC because they are necessary for cell growth; indeed, elevated polyamine concentrations have been detected in animal models of PDAC, whereas trimethylamine-N-oxide (TMAO) and its derivatives could be able to influence indirectly the onset of PDAC, promoting metabolic syndrome, obesity, and a chronic subacute inflammatory state [17,18,19]. Short-chain fatty acids (SCFAs), in particular butyrate, could modulate immunoregulation, promoting antimicrobial peptide (AMP) production in pancreatic cells, which results in a pro-inflammatory status [20]. Moreover, decreased AMP secretion in the gastroenteric tract has appeared to induce bacterial overgrowth and dysbiosis in the gut [15]. Other characteristic microbial mechanisms that could have a part in PDAC oncogenesis are suggested in *Hp* infections or supposed in *Malassezia* spp. overgrowth [21,22,23].

Besides oncogenesis, several studies have suggested a role of microbiota in PDAC locoregional and systemic therapies such as pancreatic biliary drainage (PBD), conventional gemcitabine and platinum-based chemotherapy, or immunotherapy and radiation, adding emphasis to PDCA prognosis modifications and microbiota dysbiosis [24,25]. Interestingly, animal models have hypothesized a microbiota modulation with pre-probiotics, antibiotic therapy, and fecal transplantation (FMT to improve PDAC management, but more data are necessary to confirm results) [26,27].

All this evidence now supports a role for the microbiota in the whole PDAC story. The intent of our narrative review is to report the latest literature in this regard to guide clinicians and future research in support of a better prognosis of this cancer modulated by the microbiota.

## 2. Material and Methods

**Figure 1:**
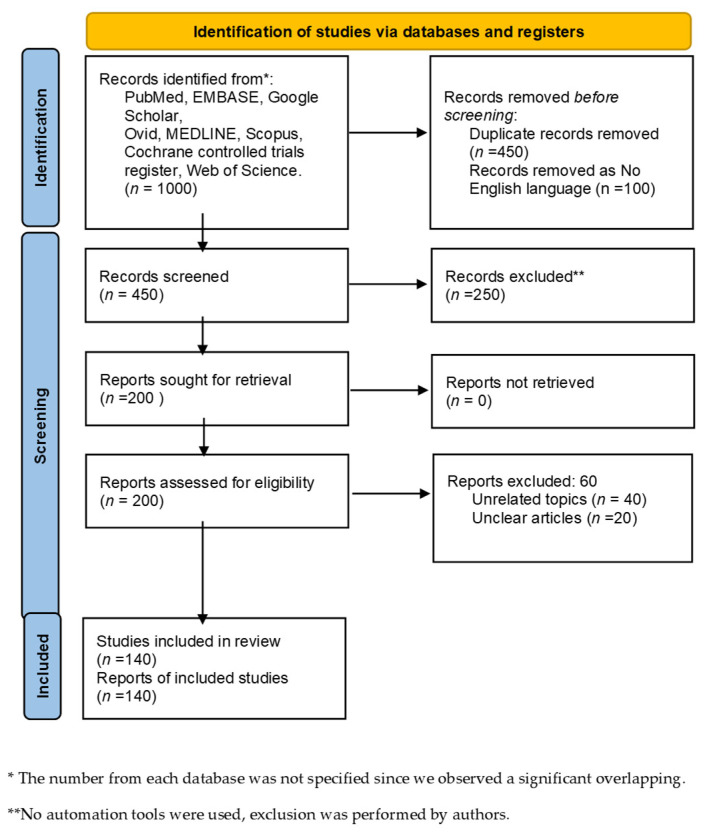
PRISMA flow diagram.

We selected articles discussing the association of microbiome and pancreatic cancer. In particular, we chose articles published in the last twenty years, focusing on the latest scientific evidence. The studies were mostly European and American, with a minority performed on eastern populations. We developed a non-systematic review article using the following electronic sources: PubMed, EMBASE, Google Scholar, Ovid, MEDLINE, Scopus, Cochrane controlled trials register, and Web of Science. We used the following single or in combination search terms: “Gastrointestinal Microbiome AND Pancreas”, “Gut microbiota AND pancreatic disease”, “precancerous pancreas AND Gut microbiome”, “PDAC AND microbiota”, “Pancreatic adenocarcinoma AND gut microbes”. We examined all the articles reporting humans’ related data (inclusion criteria), excluding works with not available full text, not in the English language, book chapters, abstracts, and articles published before 1990 (exclusion criteria). Finally, we evaluated supplementary references among articles evaluated in the first search round. A PRISMA flow diagram is reported in Figure 1.

## 3. Microbiota Pancreatic Diseases and Pancreatic Oncogenesis

The relationship between pancreatic diseases and gut microbiota (GM) is determined by the interaction among the immune system, inflammatory state, and dysbiosis [28]. Dysbiosis is characterized by a decrease in bacterial species diversity and an imbalance between bacteria with pro-inflammatory and anti-inflammatory features that can influence immunological equilibrium, e.g., an increase in segmented filamentous bacteria linked to higher levels of Th1 and Th17 cells instead of a decrease in SCFA-producing bacteria connected to higher levels of Treg cells [29]. Moreover, this compromises the integrity of the mucosal barrier with subsequent bacterial translocation, which causes the development of gastrointestinal diseases, including pancreatic diseases [30].

Normally the GM and the immune system work in symbiosis to maintain human body homeostasis, regulating the processes of cell proliferation and the vascularization, as well as blocking the excessive growth of pathogens [31,32,33,34,35]. Once homeostasis is interrupted, some microorganisms appear to be able to translocate and colonize the pancreas through the gastrointestinal lumen and blood circulation, inducing the activation of the pattern recognition receptors (PRR) of the innate immune system that are present in pancreatic acinar cells [36,37]. Secretion of pro-inflammatory cytokines and activation of the immune system is subsequently triggered. As a final result, an inflammatory process is created that, over time, may be capable of initiating pancreatic oncogenesis [38,39,40].

The role of inflammation in creating a favorable environment for the onset of cancer has been widely confirmed over the years, but the underlying molecular mechanisms are still unclear [41]. Ren et al. confirmed the role of chronic inflammation and oxidative damage in the development of pancreatic cancer. They compared the microbiota in 57 healthy people and 85 pancreatic cancer patients, highlighting in the latter an increase in lipopolysaccharide (LPS)-producing bacteria (including *Prevotella*; *Hallella*; *Enterobacter*; and other pathogens such as *Veillonella*, *Klebsiella*, and *Selenomonas)*. In parallel, a reduction of different commensals and butyrate-producing bacteria was highlighted. Evidence of an increase in LPS-producing bacteria confirms the role of dysbiosis and oxidative damage in inducing chronic inflammation through the production of pro-inflammatory cytokines and activation of the NF-kB pathway [42].

In addition to the role of pro-inflammatory cytokines, several molecular alterations, such as oncogenic mutations, appear to be involved in inflammation-mediated carcinogenesis [43].

A key role seems to be played by the Kirsten rat sarcoma (KRAS) oncogenic mutation, which is present in 90% of pancreatic cancers and appears to be promoted by the inflammatory state and by GM changes [44]. The oncogenic activation of KRAS requires the overstimulation of the LPS-driven inflammation of Gram-negative intestinal bacteria. LPS binds specifically to the TLR of pancreatic acinar cells, causing inflammation and systemic oncogenesis [45]. All this confirms the role of dysbiosis in the development of cancer through immune system activation [15,46]. However, this mutation alone would not seem sufficient to generate the development of the disease, and further triggers, which are not yet well defined, are likely needed [47].

Given the importance of the inflammatory state in promoting carcinogenesis and the action of GM in triggering this mechanism, over the years, studies have tried to understand the role of GM in promoting the development of pancreatic diseases associated with a pre-cancerous state. Table 1 reports main evidence on microbiota changes and the relative development of pancreatic disease.

### 3.1. Microbiota and Chronic Pancreatitis (CP)

CP is a fibro-inflammatory disease characterized by progressive destruction of the pancreas acinar and islet cells that are replaced by fibrous scar tissue. The perpetuation of the inflammatory stimulus causes progressive and irreversible damage to the pancreatic tissue that puts patients at risk of developing pancreatic cancer. The causes of CP are numerous, and the most common are alcohol; smoking; and metabolic, autoimmune, and genetic diseases; however, the pathogenesis of CP still remains unclear today [48].

The disease manifests with a highly variable clinical picture and evolution, which over time leads to the loss of the exocrine and endocrine function of the pancreas. Exocrine pancreatic insufficiency (EPI) manifests itself with symptoms such as diarrhea, flatulence, and abdominal bloating that may be caused by a state of dysbiosis of the small intestine [49,50]. In fact, it is well known that roughly one-third of CP patients undergo the development of small intestinal bacterial overgrowth (SIBO), a syndrome characterized by excessive growth of GM that leads to an immeasurable fermentation and inflammation of the small intestine [51]. The high frequency of SIBO in CP patients appears to be a consequence of reduced intestinal motility, reduced pancreatic synthesis of AMP, impaired formation of chyme in the lumen of the intestine, and reduced alkalization resulting from the poor pancreatic secretion of bicarbonate. This bacterial overgrowth in the small intestine is thought to exacerbate EPI and underlie the symptoms, malnutrition, and morbidity of CP patients [40,52].

In CP patients, the excessive growth of GM can create a favorable substrate for the subsequent formation of pancreatic cancer; this is both through the progressive inflammatory stimulus and the induction of molecular alterations. In fact, SIBO appears to be able to induce the mutation of the KRAS gene, highlighted in some CP patients, by means of the inflammatory response guided by LPS. This would lead to the subsequent activation of the NF-Kb signaling pathway, resulting in chronic inflammation and oxidative damage [39,53].

Given a clear role of the exocrine pancreas in the regulation of GM, studies attempted to analyze the microbiota composition in CP patients; however, the available data are still insufficient and not conclusive.

Frost et al. analyzed the GM composition and diversity in CP patients, and they found strong dysbiosis with reduced GM diversity that appeared independent of exocrine pancreatic function. In detail, *Enterococcus* overgrowth, an opportunistic pathogen that makes the CP patient at greater risk of systemic infections, was observed. The study also highlighted an abundance of facultative pathogens, such as *Streptococcus* and *Escherichia-Shigella*, and a reduction of *Faecalibacterium* and *Fusicatenibacter*, bacteria that exert an important anti-inflammatory action through the production of SCFA [49,54].

A study by Zhou et al. compared the fecal microbiota of 71 CP patients with that of 69 healthy patients. The study showed a reduction in *Firmicutes* and *Actinobacteria* and an increase in *Proteobacteria* phylum in CP patients. Furthermore, the *Eubacterium rectale* group, *Coprococcus*, *Sutterella*, and the *Eubacterium ruminantium* group were predominant in CP patients associated with EPI, while in those without exocrine insufficiency, the predominant genera were *Pseudomonas*, *Fusobacterium*, and the *Ruminococcus gnavus* group [55].

Jandhyala et al. analyzed the composition of the GM of 30 CP patients by comparing it with that of 10 healthy controls. The study showed in CP patients, especially in those with endocrine insufficiency and EPI, an important reduction of *Faecalibacterium prausnitzii*, a commensal producer of SCFA that performs important anti-inflammatory functions. Therefore, *Faecalibacterium prausnitzii* would seem to represent a factor capable of promoting the onset of diabetes and its worse progression. Similarly, a reduction in *Bifidobacterium* and *Ruminococcus bromii* was found, also responsible for an impaired glucose metabolism [52].

*Enterococcus faecalis* infection also appears to be involved in the progression of tissue damage in CP patients, favoring the subsequent development of pancreatic cancer [56].

Given the difficulty in analyzing pancreatic tissue, much of the evidence supporting the role of dysbiosis in the pathogenesis of CP comes from studies on animal models. Wu et al. evaluated the intestine microbiota in mice with cerulein-induced CP and confirmed a reduction in bacterial diversity, with lower levels of *Firmicutes* and higher levels of *Bacteroidetes*, *Actinobacteria*, and *Verrucomicrobia* [57]. Other studies, always performed on animal models, highlighted the beneficial role of enzyme replacement therapy in dysbiosis as it is able to reduce the presence of pathogens and promote the growth of bacteria capable of restoring the intestinal barrier, such as *Akkermansia muciniphila* and *Lactobacillus reuteri* [58].

Therefore, the studies underline the role of intestinal dysbiosis in the context of CP. On this basis, the identification of GM composition and the regulatory mechanisms would allow for the improvement of the knowledge of the CP pathogenesis, aiming to propose new therapeutic options. However, the data collected thus far are still insufficient, and further studies are necessary.

### 3.2. Microbiota and Autoimmune Pancreatitis (AIP)

AIP is a form of chronic pancreatitis sustained by a fibro-inflammatory process on an autoimmune basis, characterized by inflammatory lymphoplasmacytic infiltrate, fibrosis, and consequent organ dysfunction. The activated lymphocyte infiltration is mainly localized around the pancreatic ducts with subsequent periductal fibrosis that obliterates the lumen and causes an obstruction to the outflow of the pancreatic secretion. Two types of AIP are recognized: (1) type 1, characterized by high serum levels of IgG4 immunoglobulins (IgG4-RD), systemic involvement, extra pancreatic lesions, and a histopathological pattern of lymphoplasmacytic sclerosing pancreatitis; (2) type 2, characterized by a histopathological pattern of idiopathic ductocentric pancreatitis, in the absence of systemic involvement and without extra pancreatic lesions [59].

Although genetic factors are considered behind AIP, the pathogenesis of the disease remains unknown. However, even in this context, a strong correlation between microbiota, the innate immune system, and autoimmune diseases is emerging.

In fact, although AIP and IgG4-RD are characterized by a production of IgG4 Ab, with involvement of adaptive immunity, recent studies highlight the role of innate immunity in the development of the disease [60], as demonstrated by an increased expression of TLR in the pancreas of AIP patients [46,61]. GM may contribute to the activation of the innate immune system. In fact, numerous studies have shown that the activation of the immune system in AIP is a consequence of some microbial antigens and that LPS of Gram-negative bacteria can activate the immune response through TLRs. There are several TLRs involved in the development of AIP (TLR2, TLR3, TLR4, TLR5, and TLR7) and, among these, the most implicated are TLR3 and TLR7 [45,62]. All this induces a subsequent activation of antigen-presenting cells (APC), such as M2 macrophages and pancreatic dendritic cells (pDC), which ends with the triggering of pro-inflammatory cytokine responses.

Therefore, this underlines the crucial role of GM in the etiopathogenesis of AIP. In support of this, there are mainly studies performed on mice models. In detail, some works demonstrated the role of *E. coli* in the pathogenesis of the disease: through the inoculation of microbial agents on mice models, it has been shown that *E. coli* would be able to induce pathophysiological alterations typical of AIP and a parallel increase in serum IgG against *E. coli* not evident in healthy controls [63,64].

Other bacteria potentially involved in the AIP pathogenesis appear to be *Bifidobacterium*, *Fusobacterium*, and *Klebsiella* spp., but data supporting this thesis are not sufficient [11,65].

Other studies, carried out on mice models, also support the role of the microbiota in the pathogenesis of AIP. They demonstrated that the therapy with broad-spectrum antibiotics can prevent the AIP development by reducing the accumulation of the APC, such as pancreatic dendritic cells, in pancreatic tissue [65].

Interestingly, *Hp* gastric infection also appears to be associated with AIP. In fact, *Hp* appears to be able to trigger this pathology through the induction of autoimmunity and apoptosis by means of molecular mimicry pathways [66]. On the basis of this hypothesis, Guarneri et al. tried to identify potentially cross-reactive human and bacterial proteins, and they identified a strong homology between human carbonic anhydrase II (CA-II) and Helicobacter pylori alpha-carbonic anhydrase (HpCA). CA-II is a pancreatic epithelial enzyme whose specific serum antibodies are typical features of AIP. Therefore, these data assert the hypothesis that HP gastric infection can trigger AIP in genetically predisposed subjects [67].

Overall, these studies support the involvement of GM in the AIP pathogenesis, but likely dysbiosis alone is not sufficient to trigger the disease.

### 3.3. Microbiota and Pancreatic Cystic Neoplasms (PCNs)

PCNs represent a clinically complex entity and they are characterized by variable biological behavior. PCNs are divided into various types: serous cystadenoma (SCA), mucinous cystic neoplasm (MCN), intraductal papillary mucinous neoplasm (IPMN), solid-pseudopapillary neoplasms (SPN), and cystic pancreatic neuroendocrine tumors [68]. These cysts have a potential for neoplastic transformation, with a greater risk for MCN, especially for IPMN. The latter are the most common PCNs, and they are epithelial cysts, characterized by the proliferation of mucinous cells that create papillary projections within the pancreatic ducts. They can present a highly variable biological aggressiveness, ranging from low-grade dysplasia (LGD) to high-grade dysplasia (HGD) up to transformation into invasive carcinoma [69,70].

Given the risk of neoplastic transformation, proper management of PCNs is essential to prevent the development of pancreatic cancer [71]. In recent years, many studies focused on cystic fluid analysis to identify molecular and oncogenic biomarkers underlying the differentiation and transformation of NCPs. In recent years, the attention to GM is constantly increasing, and many studies are trying to understand whether the presence of bacteria inside the pancreatic cystic fluid can promote the formation or transformation of these cysts.

Li et al. studied the pancreatic cyst fluid, obtained by endoscopy, to evaluate the presence of bacterial DNA and to analyze the kinds of bacteria present inside it. The study highlighted the presence of a bacterial ecosystem consisting mainly of *Bacteroides* spp., *Escherichia/Shigella* spp., *Acidaminococcus* spp., and the less abundant *Staphylococcus* spp. and *Fusobacterium* spp. Finally, *Hp* was marginally detected in the cystic fluid. Therefore, these results underlined the presence of bacteria with already known pathogenic functions in the gastrointestinal system that could therefore be involved in the development of pancreatic carcinogenesis [72].

Similarly, Gaiser et al. analyzed the cystic fluid of 105 patients undergoing pancreatic surgery for suspected PCN. The study found higher levels of intracystic bacterial DNA and pro-inflammatory cytokine IL-1β in IPMN with HGD and IPMN with cancer compared to other types of PCN. In contrast, non-IPMN cysts, i.e., SCNs and MCNs, were low in bacterial DNA and IL-1β. This could probably be explained by the lack of direct communication of these cysts with the intestine through the pancreatic ductal system. In this study, for the first time, the amount of bacterial DNA was correlated with the severity of the neoplastic grade of IPMN [73]. Among the bacteria identified in the cystic fluid, the analysis showed that there were those typical of the oral cavity involved in the development of periodontitis and gingivitis, such us *G. adjacent*, *F. nucleatum*, *P. micra*, *E. corrodens*, *H. parahaemolyticus*, *A. odontolyticus*, *P. melaninogenica*, and *Campylobacter* spp. [74]. Among these, *F. nucleatum* has been isolated in abundance in the cystic fluid of IPMN with HGD. This would be consistent with previous recognition of *F. nucleatum* as an important oncobacterium [75]. The oncogenic role of *F. nucleatum* was also demonstrated by Alkhaaran et al., who highlighted that patients with more severe IPMN had higher *F. nucleatum* IgG and IgA salivary levels [76]. Further research is therefore needed to clarify the early involvement of this bacterium in pancreatic carcinogenesis.

In a pilot study, Olson et al. analyzed differences in the oral microbiota in patients with PDAC and IPMN, as well as in healthy controls. The study found that patients with PDAC had higher abundances of *Firmicutes* and related taxa, while healthy controls had higher proportions of Proteobacteria and related taxa. On the contrary, no significant differences were found between the PDAC and IPMN groups, where the results obtained were similar [77].

However, it should be emphasized that being faced with patients undergoing invasive endoscopic procedures, it is not possible to exclude the possible presence of oral bacterial translocation in the pancreas during the endoscopic procedure itself. To date, there has been no clear difference in intracystic bacterial composition between patients undergoing invasive endoscopic procedures or not. Therefore, the results obtained so far would support a possible role of the microbiota of pancreatic cysts in promoting local neoplastic transformation. Further studies are needed to clarify the mechanisms underlying this process and to guide the management of PNCs, especially in the follow-up.

## 4. Microbiota and Pancreatic Cancer (PC)

The human microbiota is represented by 10–100 trillion microorganisms (archeobacteria, bacteria, fungi, and viruses) inhabiting our body and in particular the gut. A relationship between dysbiosis and human pathology seemed to be linked to a lower bacterial species diversity and an imbalance between bacteria with pro-inflammatory and anti-inflammatory features but, to date, it remains difficult to highlight common modifications and mechanisms for every disease [78,79]. In the literature, several microbiome and metabolome alterations are hypothesized to have a key role in this relationship:

- α-diversity reduction, which leads to lower variability in human microbiota core and interactions.
- *Bacteroides*–*Firmicutes* ratio imbalance with an abundance of pathogenic bacteria and loss of physiological ones.
- Pro-inflammatory and anti-inflammatory metabolite production imbalance with a decrease in SCFA production instead of an increase in TMAO and its processing of its derivatives.

To date, mounting evidence has suggested an independent relationship between microbiota dysbiosis and PDAC development via chronic flogosis stimulation and oncogene (e.g., KRAS) upregulation, as mentioned below in the text [1]. Although several studies have established the presence of microbiota within the pancreas, the exact mechanisms by which microbiota could reach the pancreas are still unknown. It is possible that microbiota could migrate from the upper and lower gastroenteric tract to the pancreas through the major ampulla in the duodenum or via the mesenteric venous and lymphatic system from the gut [16,17]. In the last assumption, a defective intestinal permeability (e.g., caderine unit downregulation) and microbe translocation are supposed to be the result.

The first commensals linked to a possible role in human pancreatic cancer were part of the oral microbiome [2,80,81]. It is now established that the association between poor oral health and the development of PC is well supported in that inflammation of the gingiva, periodontal disease, and tooth loss represent independent risk factors for PDAC [82,83,84,85,86]. Farrel et al. compared the salivary microbiota of PDAC patients and healthy control subjects [87]. Their study found 31 increased bacterial species/clusters and 25 decreased ones in the salivary samples of patients with PDAC in comparison to those of the healthy controls. Furthermore, there was a variation of oral microbes, with an association between oral pathogens *Porphyromonas gingivalis*, *Fusobacterium*, *Neisseria elongata*, *Streptococcus mitis*, and PDAC. Fan et al. recruited 361 PDAC patients and 371 healthy controls from prospective cohort studies to compare their pre-diagnostic oral wash samples and characterize their oral microbiota [80]. They also detected an increased carriage of periodontal bacteria such as *P. gingivalis* and *Aggregatibacter actinomycetemcomitans*, and a decreased one of *Fusobacteria* and *Leptotrichia* unlikely due to potential confounders, e.g., smoking or alcohol abuse.

Indeed, in the near future, such variations in salivary microbiota from patients with PDAC could support the possibility of using salivary microbial biomarkers for systemic disease prediction. In this context, *P. gingivalis* and *A. actinomycetemcomitans* were associated with a higher risk of PC, while conflicting evidence has been found regarding *Fusobacteria* genera that were decreased in some studies but increased in others [88]. To examine the relationship between host immune response, oral microbes, and pancreatic cancer risk, Michaud et al. measured antibodies to oral bacteria in blood samples from 405 PC cases, before their diagnosis, and 416 matched healthy controls [87]. They found that patients with high levels of *P. gingivalis* antibodies (>200 ng/mL) had a twofold higher risk of PC (OR 2.14; 95% Cl 1.05, *p* = 0.05). Detection of antibodies against these oral bacteria could be utilized as a biomarker to identify people with a high risk of PC, but the impact of *P. gingivalis* on pancreatic oncogenesis, if any, to date is unknown. Whereas the previous studies relied on the oral microbiome, increasing evidence has associated changes in the gut microbiota with pancreatic diseases as well [20]. It is thus natural to hypothesize that alteration of the gut microbiota could also be associated with PDAC and could provide a potential screening mechanism in the near future. To investigate a potential link between the gut microbiota and PDAC, different studies have characterized the fecal microbiota to elucidate any gut microbiota alterations in patients with PDAC. Ren et al. collected fecal samples of 85 PDAC patients and 57 matched healthy controls to analyze gut microbiota differences [42]. The results showed a decreased α-diversity and a unique microbial profile characterized by an increased abundance of potential pathogens and a decrease in beneficial bacteria when compared to healthy controls. The fecal samples of PDAC patients had an increase in LPS-producing bacteria (e.g., *Prevotella*, *Hallella*, *Enterobacter*, *Veillonella*, *Klebsiella*, and *Selenomonas*) that are often associated with chronic flogosis stimulation and increased risk for cancer, as well as a decrease in SCFA-producing bacteria that has demonstrated anti-inflammatory features. In particular, in animal models, LPS have been shown to induce TLR signaling cascade (e.g., TLR2, TLR9), NF-kB, and KRAS pathway upregulation, all involved in flogosis stimulation and carcinogenesis [89]. LPS seemed also to improve lymphocyte infiltration and PD-L1 expression via TLR4, which could be involved in tumor immune escape [90]. In other animal models, SCFAs, and above all butyrate, could modulate immunoregulation, promoting antimicrobial peptide (AMP) production in pancreatic cells that resulted in a macrophage switch from an inflammatory (M1) to a regulatory (M2) phenotype and T-reg cells [20]. Decreased AMP secretion (e.g., cathelicidin-related antimicrobial peptide or CRAMP) in the gastroenteric tract has seemed to induce bacterial overgrowth and dysbiosis in the gut [15]. Furthermore, experimental evidence in mouse models has suggested that gut microbiota accelerates pancreatic cancer development and produces an overall suppression of antitumor immunity by increasing myeloid-derived suppressor cell infiltration and by reducing antitumor cytotoxic CD8+ T cells [15].

Other microbial mechanisms could have a role in PDAC oncogenesis. Satoru et al. examined the effects of *Hp* infection on human PDAC cell lines [20]. The study found high IL-8 and vascular endothelial growth factor (VEGF) secretion levels in tumor cells co-cultured with *Hp*. Moreover, several proliferation factors such as NF-kB, activator protein-1 (AP-1), serum response element (SRE), and cytotoxin-associated gene A (CagA) protein expression were increased in *Hp* co-culture tumor cells. Raderer et al. detected high seropositivity in blood samples from patients with PDAC and GAC compared to healthy controls, as seen for *P. gingivalis* [36,91]. On the basis of the above studies, it could be possible to validate a relationship between *Hp* infection and the development of PDAC; however, others have not confirmed these data [92].

Kazmierczak-Siedlecka et al. also hypothesized several *Fungi* genera infections such as *Candida*, *Malassezia* spp., and *Trichosporon* as co-factors in PDAC onset. In this review, several molecular mechanisms are suggested to be involved in PDAC oncogenesis, e.g., augmented activation of mannose-binding lectin (MBL) way, enhanced pro-inflammatory cytokine IL-6 activation, and carcinogenic metabolite production. Lastly, *HBV* and *HCV* in the TLR ion seemed to improve PDAC risk [93,94,95].

Microbiota could have also a role in PDAC drug resistance, improving fibrosis and impaired drug delivery in tumor mass by activating cancer-associated fibroblasts (CAFs) via TLR9 ligation [89]. Interestingly, a murine model showed an augmented gemcitabine response in mice with PDAC if chemotherapy was co-administered with ciprofloxacin, although confirmational data are necessary [2].

Different microbiome profiles have also been related to PDAC prognosis. Several species such as *Pseudoxanthomonas*, *Streptomyces*, and *Saccharopolyspora* have been associated with long-term survival (LTS) rates instead of *Fusobacteria* and *Rothia* bacteria that have been linked to short-term survival (STS) rates and poor prognosis [26,96].

On the basis of the above studies, it could be natural to think that the microbiota could be engaged in PDAC onset and development. Nevertheless, more investigations are needed to confirm results and determine a more precise way to deeply understand the relationship between microbiota and the development of PDAC. In particular, these data would beg further research in clinical scope to implement precision medicine purposes.

**Table 1:** Main microbiota alterations and linked modifications observed in pancreatic diseases.

Disease	Ref.	Main Microbiota Alterations	Main Modifications Linked to Microbiota Alterations
Autoimmune pancreatitis (AIP)	[63,64]	↑ *Escherichia coli*	connections with typical alterations of AIP and increase in serum IgG
	[11,65]	↑ *Bifidobacterium*, *Fusobacterium*, and *Klebsiella* spp.	antibiotics can prevent AIP development by reducing the accumulation of the APC in pancreatic tissue
	[66,67]	*↑ Helicobacter pylori*	↑ AIP development through the induction of autoimmunity and apoptosis through molecular mimicry pathways: strong homology between CA-II and HpCA
Pancreatic cystic neoplasms (PCNs)	[72]	↑ *Bacteroides* spp., *Escherichia-Shigella* spp., *Acidaminococcus* spp., *Staphylococcus* spp., *Fusobacterium* spp., *Helicobacter pylori*	
	[73,74,75]	*↑* levels of intracystic bacterial DNA *↑ Fusobacterium nucleatum*, *Parvimonas micra*, *Eikenella corrodens*, *Hemophilus parahaemolyticus*, *Actinomyces odontolyticus, Prevotella melaninogenica*, and *Campylobacter* spp.	↑ pro-inflammatory cytokine IL-1β in IPMN with HGD and IPMN; in contrast, non-IPMN cysts were low in bacterial DNA and IL-1β
	[77]	↑ *Firmicutes* with related taxa and ↓ *Proteobacteria* with related taxa in patients with IPMN and PDAC compared to healthy controls	
Pancreatic ductal adenocarcinoma (PDAC)	[87]	↑ 31 bacterial species/clusters and ↓ 25 ones belong to *Firmicutes*, *Proteobacteria*, *Actinobacteria*, and *CFB group bacteria* philia in patients with PDAC compared to healthy controls	innate and acquired immunity gene upregulation through ↑ TLR-signaling, ↑ NF-Kb activation, ↑ chronic flogosis, and cancerogenesis
	[80]	↑ Porphyramonas gingivalis and *Aggregatibacter actinomycetecomitans* ↓ *Fusobacteria* and *Leptotrichi*	connections between periodontal pathogens and increased risk of pancreatic cancer; associations unlikely due to smoking or other potential confounders
	[88]	↑ *Fusobacteria*	
	[81]	↑ *Porphyramonas gingivalis* antibodies (>200 ng/mL) linked to a higher risk of PC	
	[42,43,44]	↓ *α*-diversity in microbiota profile ↑ LPS-producing bacteria (*Prevotella*, *Hallella*, *Enterobacter*, *Veillonella*, *Klebsiella*, and *Selenomonas*) ↓ SCFA-producing bacteria	↑ chronic inflammation through the production of pro-inflammatory cytokines and activation of the NF-kB pathway ↑ KRAS activation binding TLR of pancreatic acinar cells, increasing inflammation and cancerogenesis
	[15,97]	↑ SCFA-producing bacteria	↑ AMP production in pancreatic cells resulted in a macrophage switch from an inflammatory (M1) to a regulatory (M2) phenotype and T-reg cells; ↓ AMP secretion in the gastroenteric tract seemed to induce bacterial overgrowth and dysbiosis in the gut and pancreatic cancer development by increasing myeloid-derived suppressor cell infiltration and by reducing antitumor cytotoxic CD8+ T cells
	[20,91]	effects of *Helicobacter pylori* infection in PDAC cell lines ↑ Helicobacter pylori antibodies in patients with PDAC and GAC compared to healthy controls	↑ IL-8, VEGF, NF-kB, AP-1, SRE, and CagA expression in tumor cells co-cultured with *Helicobacter pylori*
	[93]	↑ *Candida*, *Malassezia* spp., and *Trichosporon*	↑ activation of MBL way, ↑ IL-6 and carcinogenic metabolite production

## 5. Microbiota among the Different Phases of Locoregional Treatment

Nowadays, there is a rising interest in the characterization and activity of gut microbiota (bacterial as well as viral and fungal) in patients affected by pancreatic cancer (PC), with particular focus on the comprehension of its modifications following therapeutic interventions and its potential role in the management of this disease during the different phases of treatment.

### 5.1. Biliopancreatic Endoscopy and Surgery

The cornerstone of therapy for PC is represented by surgery, as a complete resection represents a potentially curative option for PC. Despite technical progress and the high expertise in referral centers for hepatobiliary–pancreatic surgery, rates of postoperative morbidity and mortality are relatively high, and postoperative complications have been related to tumor recurrence and worse survival rate in PC [98,99].

Since patients with head PC often suffer from obstructive jaundice, percutaneous or endoscopic biliary stenting procedures become essential prior to surgical evaluation, particularly in the era favoring total neoadjuvant therapy [100]. This trend of biliary drainage at diagnosis has been increasing from 30% in 1992–1995 to 59% in 2000–2007 in the United States [24].

Several authors attempted to establish whether there is a relationship between preoperative biliary drainage (PBD) and morbidity after pancreaticoduodenectomy (PD) for PC. It was supposed that PBD can lead to ascending microbial migration and consequently to an increased risk of infections, in particular those of surgical site (SSI) cholangitis till sepsis. The literature is now abundant with the hypothesis that patients undergoing PBD have an increased rate of positive intraoperative bile cultures and an increased infection-related morbidity and mortality rate.

Some years ago, researchers validated a novel risk score to predict SSI after PD across 679 PD patients from a very high volume center, with a total of 117 (17.2%); univariate analysis revealed seven risk factors, while on multivariate analysis, PBD and neoadjuvant chemotherapy were independent predictors of SSI [101].

Many studies have shown that biliary stents lead to higher SSI rates. Barreto et al. compared the characteristics of patients who underwent PD and developed SSIs108, reporting that patients who received a PBD were more likely to have SSI than those who did not receive a preoperative stent.

In 2006, Cortes et al. published a case–control study on 79 consecutive patients with periampullary tumors who underwent PD with routine bile culture [102]. Bile contamination, through previous endoscopic intervention, had a remarkable impact on immediate complications of PD, with a higher rate of infections, especially wound and intraabdominal abscesses. They also demonstrated a notable correlation between positive bile culture, length of stay in an intensive care unit, and rate of prolonged postoperative antibiotic therapy. Bile contamination was present mainly in patients who underwent preoperative endoscopic procedures.

In 2011, Morris-Stiff et al. demonstrated that, in 280 patients undergoing PD for periampullary malignancies, pre-operative stent insertion was associated with increased morbidity but not mortality, and this was higher for stents placed endoscopically rather than percutaneously [103].

In 2018, a systematic review and meta-analysis was published, including a total of 28 studies (8523 patients) about the impact of bacterobilia on morbidity and postoperative management after PD [104]. The median incidence of bacterobilia was 58%. The most frequently isolated bacteria species were *Enterococcus* (51%), *Klebsiella* (28%), and *E. coli* (27%). PBD was significantly correlated to bacterobilia, and in this cluster of patients, the incidence of SSI was significantly greater. Matching bacteria in bile and the infectious sites were found in 48% of the cases.

A single-center retrospective study was conducted in 2017 among patients with obstructive jaundice due to periampullary cancer in order to analyze the effect of PBD (with plastic or metal stent positioning) on the microbiome of the biliary system and on postoperative outcomes [25]. They stated that stent placement can affect the composition of biliary microbiota, and colonization of microbes from the biliary tract, next to the pancreas, may influence the proliferation of intra-tumor microbiota. The biliary microbiome of patients after PBD was mainly characterized by *Enterococcus* species (*E. faecalis*, *E. faecium*, *Enterobacter cloacae*), with a significant statistical prevalence of antibiotic resistance. Among post-operative complications, only the frequency of wound infection was significantly related to a positive bile culture, especially in the presence of *E. faecium* and *Citrobacter* species in the bile. These investigators also supposed that plastic stents could lead to greater alterations in biliary microbiota because they have a greater risk of recurrent obstruction and lower patency compared to metal ones.

Similarly, in the retrospective study of Stecca et al. on 128 patients undergoing PD with or without PBD [105], bacterobilia was significantly higher in the stented group, with a 100% infection rate compared to 8.3% for early surgery. The most frequently accountable for resistant bacterobilia were as follows: *Escherichia* spp., *Enterobacter* spp., *Klebsiella* spp., *Candida* spp., and *Enterococcus* spp. [106,107].

Sudo et al. reported an increase in the proportion of patients with bacteria in the bile after PBD compared with those having no drainage (78 versus 36 percent, respectively) [108]. A higher frequency of a polymicrobial spectrum was found patients after PBD, including a higher prevalence of *Klebsiella*, *Enterobacter*, *Citrobacter*, *Enterococcus*, *Streptococcus*, and *Staphylococcus*. Nomura et al. also found that the presence of *Enterococci* in the bile was associated with increased complications after surgery [109].

Nadeem et al. also confirmed an increased rate of Gram-negative anaerobic bacteria on bile cultures (*Klebsiella*, *E. coli*, *Enterobacter*) [109]. This finding is in agreement with previously published data, and it may be explained by a greater exposure to cephalosporins during episodes of stent-related cholangitis and/or cholecystitis [110,111].

Very recently, Nalluri et al. demonstrated that bacterial migration to tumoral tissue was more liable in patients with the involvement of pancreatic head and those who underwent the Whipple procedure [112]. This could be explainable by the contiguity between pancreatic head tumors, and the gastrointestinal and hepatobiliary system was also more observable. Notably, patients who had undergone PBD with stent placement registered the presence of more intra-tumoral microorganisms as well as a greater relative predominance of *Enterobacteriaceae*.

Interestingly, differently from Scheufele et al., they found that the type of biliary stent did not influence the proliferation of intratumor bacteria [25].

The impact due to the type of stent used for PBD is still debated; indeed, it appears to be important for the development of postoperative complications. In a randomized trial comparing self-expandable metal stents with plastic stents, Soderlund et al. described a median better patency for metal stents rather than plastic ones [113,114]. Moreover, Moses et al. reported an increased incidence of cholangitis after placement of plastic stents in comparison to metal stents, therefore worsening bacterial resistance due to an amplified demand for antibiotic treatment of cholangitis.

Even the very recent literature is congruently in agreement with the previous results on the topic. Bilgic et al. in 2020 described, in a retrospective series of 214 patients, that biliary stent insertion (both endoscopic or percutaneous) increased both the risks of biliary bacterial colonization and the rate of SSI [115]. The postoperative complications and duration of hospitalization were also higher in the PBD group. In 15% of patients, it was found that the same pathogen was isolated from both bile fluid and the surgical site with similar antimicrobial susceptibility, suggesting a causal effect. The most common microbes implicated in SSI were *E. coli* (29%), *Enterococcus* spp. (15%), *Klebsiella* spp. (13%), *Pseudomonas* spp. (4%), and *Candida* species (6%). Additionally, ampicillin-sulfabactam resistance was more relevant in the PBD group.

A deeper analysis was conducted by Shrader et al., who evaluated the impact of bile bacterial contamination on PC cell survival, enrolling stented and not-stented patients undergoing PD [116]. They found that most of the stented bile samples had much less anti-cancer properties and that a specific kind of bacteria usually found in the upper gastrointestinal system (*Enterococcus* and *Streptococcus*) isolated from the stented “contaminated” bile assay can modify the biological effects of bile on tumoral cell survival, in accordance with bacterial strains, cell lines, and original “sterile” bile samples. This result reflects the possibility that the dissemination of the intestinal microbiome into the biliary tree through stenting may alter the biological behavior of bile towards PC cells.

Besides infectious complications, another common issue after PD is postoperative pancreatic fistula [117]. The microbiome in bile may also influence this aspect, as reported by Ohgi et al., who identified positive intraoperative bile culture as one of the independent risk factors for postoperative pancreatic fistulas [118].

Moreover, there is emerging evidence that gastrointestinal reconstructions can influence the composition of the gut microbiota. In a mouse model undergoing Roux-en-Y gastric bypass, it was found that there was a greater load of *Bacteroidetes*, *Verrucomicrobia*, and *Proteobacteria* in stools and a transfer of the gut microbiota from treated mice to non-operated, germ-free mice, resulting in weight loss and decreased fat mass in the recipient animals, suggesting that altered gut microbiota may trigger weight loss after RYGB, concluding that microbes may impact on weight loss following surgery in patients with PC [119].

### 5.2. Radiation

Gut dysbiosis seems to contribute to a weakened effectiveness of radiation in oncological patients. Radiation is frequently considered for PC treatment in a palliative setting, but phase I/II clinical trials are currently testing radiation in combination with immunotherapies [120].

Gamma irradiation can lead to significant alteration to the gut microbiome, involving an increase in the *Alistipes* spp. (*Bacteroidetes*) and *Corynebacterium* (*Actinobacteria*) genera and a decrease in the *Prevotella* genus (*Bacteroidetes*). The *Alistipes* species, particularly *A. onderdonkii*, is downregulated in PC, and its upregulation was related to the inhibition of PC cell proliferation [121]. In the literature, it is reported that there is a suppression of the *Corynebacterium* genera instead of rising ranks of the *Prevotella* genera between PC and healthy patients—this imbalance may influence the innate and adaptive immune suppression [15,122].

These premature results led to speculation that radiation can contribute to PC cell death, not only promoting induction of DNA impairment but also modifying the composition of the gut microbiome. Although there is some advance in comprehension of the effect of radiation in PC, the correlation between response rate and gut microbiome has still not been deeply examined in PC as in other cancers, and thus further investigations need to focus on the possible improvement in response rates.

## 6. How the Microbiome Could Guide Systemic Therapy

### 6.1. Chemotherapy

The gut and intratumoral microbiome has emerged as an important factor in the treatment of PC at various levels, as it can play a role considering chemotherapy resistance, efficacy, and toxicity. The link between gut microorganisms and chemotherapy is bidirectional since they can be responsible for deep changes in the commensals’ profile.

Systemic therapy of pancreatic cancer is a cornerstone. Chemotherapy is still the main treatment adopted in cases of metastatic or locally advanced disease, whether neoadjuvant or adjuvant. It is a heavy and often poorly tolerated therapy. Gemcitabine is the main first-line drug for solid digestive tumors, including PDAC chemotherapy. There is various evidence in animal models that there is a microbiological basis for a different response to gemcitabine [123]. In particular, two studies recently enhanced these new perspectives in pancreatic cancer. Geller et al. showed an important role for intratumoral *Gammaproteobacteria*, responsible for drug resistance in 86 (76%) among 113 human PDACs. This is the first contribution demonstrating that bacteria are a component of the PDAC mass microenvironment that are able to predict therapeutic response [124].

In addition to cancer intrinsic and tumor-microenvironment-driven factors that contribute to therapy resistance, there is interesting evidence on pre-existing conditions contributing to this. A type 2 diabetes (T2D) mouse model was studied that confirmed the hypothesis that microbial dysbiosis is associated with increased resistance to chemotherapy [125].

Fluoropyrimidines such as 5-fluorouracil (5-FU), capecitabine, or TAS-1 are often co-administered with gemcitabine, leading to toxicity and their being limited by resistance. A first study conducted on mouse models in Taiwan systematically analyzed the effects and safety of *Lactobacillus* strains on 5-FU-induced mucositis and assessed positive changes in the intestinal microbiota after probiotic intervention in terms of abundance and diversity [126]. The *Caenorhabditis elegans* system was used to study how bacteria affect the response to chemotherapeutics. Garcia-Gonzalez et al. performed a genetic analysis in two bacterial species using three chemotherapeutic drugs 5-fluorouracil (5-FU), 5-fluoro-2′-deoxyuridine (FUDR), and camptothecin (CPT), finding numerous bacterial nucleotide metabolism genes that affect drug efficacy in *C. elegans* [127]. The main evidence emerged from studies on colorectal cancer models, some recently focusing on the role of *F. nucleatum* for chemoresistance by modulating autophagy [128,129,130].

Oxaliplatin is a third-generation platinum-based drug approved for PDAC first-line treatment. Dysbiosis was linked to altered response, even to this therapy. The disruption of the microbiota was shown to compromise the response of subcutaneous tumors to platinum chemotherapy in a mouse model, and furthermore microbiota mediated its effects by modulating myeloid-derived cell functions in the tumor microenvironment [131].

The second level of involvement of the microbiome in this setting is as a direct therapeutic counterpart, considering antibiotics and modulation of microbial species involved in immunoactivation. Chandra et al. recently enhanced three independent studies conducted on different PDAC models, providing evidence that the broad ablation of microbes is an effective approach to affect premalignant lesions and pancreatic cancer progression [132]. The supposition of these studies is that the microbiome promotes cancer progression by inducing immune suppression.

Sethi et al. first studied the effects of gut microbiome depletion by oral antibiotics on tumor growth in a subcutaneous and liver metastases model of pancreatic cancer, colon cancer, and melanoma. They exposed the mouse models to standardized broad-spectrum oral antibiotics (vancomycin, neomycin, metronidazole, ampicillin, and amphotericin B). The first evidence was a tumor-decreasing effect of antibiotics, requiring active participation of adaptive immunity; thereby, they linked the exposition to oral antibiotics with the balance between pro- and anti-tumor T cells [133]. Likewise, a subsequent research in mice models confirmed that modulation of the PDA microbiome to augment immunotherapy seems to be an attractive chance to improve immunotherapeutic response to cancer [15]. The role of microbiota and immune pathways is further complicated by the fact that we do not know whether it matters more for the pancreatic microenvironment or whether it acts at a distance from the gut. According to Thomas et al., normal and malignant pancreatic tissue harbors a microbiota, but while this microbiota was not able to differentiate between disease states, the presence of intestinal microbes accelerated PDAC progression, utilizing both a transgenic and xenograft mouse model of pancreatic cancer [97]. The possible use of antibiotic therapy, however, remains controversial due to the growing problem of antibiotic resistance, the need for systemic or local administration, and the non-univocal benefit in various types of tumors.

### 6.2. Immunotherapy

Growing evidence from animal models and human studies indicates that the gut microbiota may have a role not only in the occurrence of complications after pancreatic surgery but also in the effectiveness of novel targeted immunotherapies [134]. Immunotherapeutic approaches that are currently under examination for PC regard immune checkpoint inhibitors (ICIs), vaccine therapy, adoptive cell transfer, myeloid-targeted therapy, immune agonist therapy, and combinations with chemoradiotherapy or other molecularly targeted agents; in actuality, thus far, treatment with ICIs has been unsuccessful in PC [135,136,137]. While solid tumors are proven to proliferate in an immunosuppressive environment, the reasons why PC has a relatively poor response to ICI-based therapeutic approaches are not known [137].

The expression pattern of ICI (such as programmed cell death protein 1 (PD-1), programmed cell death protein ligand 1 (PDL-1), and citotoxyn T-lymphocyte-associated protein 4 (CTLA4)) in the PC microenvironment is not well understood, and several clinical trials are nowadays focusing on the characterization of the microbiome and its role in PC in the immunological field. One of these regards the use of pembrolizumab (*NCT03637803*) in association with lyophilized bacteria, showing a restricted response in otherwise ICI-refractory metastatic lung, renal, and pancreatic cancer. Another current clinical study is evaluating the combinatorial benefit of probiotics with vancomycin and nivolumab (*NCT03785210*) in patients with refractory liver and pancreatic cancer.

Tan et al. showed that a single dose of attenuated *Salmonella typhimurium* that was bioengineered to express cytolysin A markedly inhibited the growth of murine PC xeno- and orthografts. This was accompanied by the destruction of stromal cells in PC, along with enhanced infiltration with anti-tumor immune cells [138]. Similar pre-clinical studies with S. typhimurium bioengineered to express collagenase and hyaluronidase demonstrated reduced collagen fibers in PC, resulting in a reduced tumor burden and proliferation [139,140]. Bioengineered *Listeria monocytogenes* designed to express mesothelin, a PC-associated tumor antigen, induced the efficient activation of cytotoxic CD8+ T cell responses, inducing PC tumor regression [141,142].

Bacteria can exercise both positive and negative reactions on the immune response. For example, *Bacteroidetes* spp. were shown to activate Th1 immune responses, and *Listeria monocytogenes* altered tumor associated macrophages from one immunosuppressive phenotype to another. The immune reaction in oncological treatment has been shown to be improved by the inhibition of T-reg cells through *Bifidobacterium adolescentis*, *Enterococcus faecium*, *Collinsella aerofaciens*, and *Parabacteroides merdae* [143]. The gut microbiota has been shown to increase the efficacy of blockade therapy of the PD-1 protein and PD-L1 [144]. On the other hand, the anticancer immune response increased and the tumor burden was reduced by depletion of the gut microbiota through oral gavage antibiotics treatment in a mouse model of PC [145].

In 2019, Riquelme et al. compared surgically resected patients who survived more than 5 years after surgery (long-term survivors, LST) with short-term survivors (less than 5 years) to explore the role of the human tumor microbiome composition in mediating clinical outcomes of PC patients [26]. Their finding suggest that, independent of therapy, the PC tumor microbiome diversity and composition can influence immune infiltration that ultimately influences PC survival. In particular, they found a signature of three tumor bacterial taxa, namely, *Sachharopolyspora*, *Pseudoxanthomonas*, and *Streptomyces*, significantly enriched in long-term survival patients. The presence of *Bacillus clausii*, one of the top species enriched in LTS, combined with the three genus signature was highly predictive of long-term survivorship. In the future, tumor microbiome sequencing could be used to stratify patients for adjuvant trials, including microbiome interventions.

Erlotinib is a targeted immunotherapic drug sometimes prescribed in combination with chemotherapy. Gut microbiota has been proposed as a model to predict response to this kind of therapy. A recent contribution published by Heshiki et al. showed a cohort of 26 patients with various cancer types, treated either with cytotoxic or targeted chemotherapy (n = 15) or a combination of cytotoxic or targeted chemotherapy with immunotherapy (n = 11). They collected fecal samples and performed a comparison with publicly available data to evaluate whether the cancer patients presented distinct gut microbial profiles. Human gut metagenomic analysis revealed that responder patients had significantly higher microbial diversity and different microbiota compositions compared to non-responders. A machine-learning model was proposed and validated in an independent cohort to predict treatment outcomes on the basis of gut microbiota composition and functional repertoires of responders and non-responders. Specific species, *Bacteroides ovatus* and *Bacteroides xylanisolvens*, were positively correlated with treatment outcomes. Oral gavage of these responder bacteria significantly increased the efficacy of erlotinib and induced the expression of CXCL9 and IFN-γ in a murine lung cancer model. They showed that gut microbiome signatures at baseline can accurately predict cancer treatment outcome [146].

Although the greater microbial diversity could profoundly affect carcinogenesis and the development of immune anti-tumoral response through the inflammatory activation of tumor-infiltrating immune cells, its role is not entirely clear and remains to be explored specifically in PC [131,147]. Future investigations will determine if a similar mechanism can be used by the tumor microbiota to modulate the immune system by improving or impairing the immune response against the tumor [145]. Possible future scenarios are the intentional manipulation of the gut microbiota in association with therapeutic approaches [143].

### 6.3. Faecal Microbiota Transplantation (FMT)

In this perspective, FMT could be the peak of this approach in counteracting dysbiosis [144]. A single pilot study is registered (*NCT04975217*) and is actually recruiting patients with the primary objective of assessing the safety, tolerability, and feasibility of FMT in respectable PDAC patients. The intervention is scheduled as FMT during colonoscopy and FMT capsules via OS once a week for 4 weeks before surgery. They will be followed up to 6 months after surgery to determine immunological/molecular changes, as well as to assess changes in the gut, oral, and intra-tumoral microbiome. There is also emerging evidence suggesting the beneficial role of FMT to improve immunotherapeutic outcomes in cancer patients. However, their mechanisms in enhancing or attenuating the efficacy of immunotherapies need to be identified. Through FMT or supplementation with certain prebiotics, probiotics, or antibiotics, the gut microbial composition could be manipulated to enhance host anticancer immunity and combat drug resistance. Moreover, the gut microbiota could be used as a biomarker for drug efficacy, treatment response, and drug side effects. Table 2 summarizes current evidence on microbiota and the management of systemic therapy of PDAC.

## 7. Conclusions

Pancreatic cancer still carries a severe prognosis, with few cases of resectable disease at diagnosis. The possibility that the intestinal and extra-intestinal microbiota may change the management of this disease is therefore arousing great interest in the scientific community. As previously summarized, mounting evidence has suggested a relationship between human microbiota and different PDAC phases: pancreatic diseases with increased risk for PC, carcinogenesis, and locoregional and systemic therapy. However, almost all available studies are mice models, and few clinical available trials require a larger cohort to achieve an adequate bias control. Accordingly, these data are needed for further research, particularly as they relate to mechanisms, human diversity, and the implementation of precision medicine.

## Figures and Tables

**Table 2:** Microbiota influence on locoregional and systemic treatment.

Treatment	Ref.	Microbiota in Locoregional and Systemic Treatment
Biliary drainage and surgery	[25,102,103,106,108,109,116,118]	PBD can lead to ascending microbial migration and consequently to an increased risk of infections, fistulas, cholangitis, and sepsis. This eventuality could increase infection-related morbidity and mortality rate after surgery. PBD can affect the composition of biliary microbiota. In particular, ↑ *E. faecalis*, *E. faecium*, and *Enterobacter cloacae* are linked to antibiotic resistance that could complicate post-surgery infection treatment. PBD can influence the proliferation of intra-tumor microbiota as well as a greater relative ↑ *Enterobacteriaceae.* It is not clear yet if a different type of biliary stent (e.g., plastic or metal) could differently influence post-PBD infection risk. Plastic stents seem to be linked to a lower median patency rate and, as consequence, are a major bacterial contamination risk.
Radiation	[121,122]	Radiation can lead to significant alteration to the gut microbiome: ↑ *Alistipes* spp. and *Corynebacterium* genera and ↓ in the *Prevotella* genus. *Alistipes onderdonkii* upregulation could be related to the inhibition of PC proliferation cells. Radiation can contribute to PC cell death not only by promoting the induction of DNA impairment but also modifying the composition of the gut microbiome.
Chemotherapy	[15,97,124,131,132,133]	Bacteria are a component of the PDAC mass microenvironment that could influence therapeutic response (e.g., *Gammaproteobacteria).* Ablation of microbes with antibiotics seems to be an effective approach to affect premalignant lesions and pancreatic cancer progression. These studies suppose that the microbiome can promote cancer progression by inducing immune suppression.
Immunotherapy	[138,139,141,142,143,144,145]	A single dose of attenuated *Salmonella typhimurium* bioengineered to express cytolysin A inhibited the growth of murine PC xeno- and orthografts. *Salmonella typhimurium* bioengineered to express collagenase and hyaluronidase reduced collagen fibers in PC, resulting in a reduced tumor burden and proliferation. *Listeria monocytogenes* bioengineered to express mesothelin induced the efficient activation of cytotoxic CD8+ T cell responses and PC tumor regression. Oncological treatment was shown to be improved by the inhibition of T-reg cells through *Bifidobacterium adolescentis*, *Enterococcus faecium*, *Collinsella aerofaciens*, and *Parabacteroides merdae*. Microbiota were shown to increase the efficacy of PD-1 and PD-L1 blockade therapy. Depletion of the gut microbiota through oral gavage antibiotics increased anticancer immune response and reduced the tumor burden in a mouse model of PC. Human gut metagenomic analysis revealed that responder patients had significantly higher microbial diversity and different microbiota compositions compared to non-responders. In particular, *Bacteroides ovatus* and *Bacteroides xylanisolvens* were positively correlated with treatment outcomes.
Fecal microbiota transplantation (FMT)	[144]	A single pilot study (*NCT04975217*) is recruiting patients to assess the safety, tolerability, and feasibility of FMT in respectable PDAC patients. There is emerging evidence suggesting the beneficial role of FMT to improve immunotherapeutic outcomes in cancer patients.

## Data Availability

Not applicable.
